# RRT-Based Incremental Path Search over Incrementally Revealed 3D Scene Graphs

**DOI:** 10.3390/s26216800

**Published:** 2026-10-28

**Authors:** Haobin Liang, Hailong Pei

**Affiliations:** The Key Laboratory of Autonomous Systems and Network Control, Ministry of Education, Guangdong Engineering Technology Research Center of Unmanned Aerial Vehicle Systems, South China University of Technology, Guangzhou 510641, China; auhb.liang@mail.scut.edu.cn

**Keywords:** 3D scene graph, path searching, Rapidly-exploring Random Tree

## Abstract

Graph-based path planning commonly operates on an available topological representation, but robotic exploration may require searching for feasible paths while the environment graph is progressively revealed. This paper proposes MSG-RRT (Multi-Strategy Scene-Graph RRT), an RRT-based method for first-feasible-path discovery over incrementally revealed 3D scene graph topology. MSG-RRT operates on the discrete spatial-topological structure of the Places layer and combines goal-biased graph-node sampling with topology-constrained, goal-aware steering. Tree expansion is coupled with simulated local graph revelation, allowing newly revealed topology to participate in subsequent search. Experiments on a scene graph generated from the uHumans2 environment show that MSG-RRT reduces the average number of search iterations from 2185.45 to 683.90 and runtime from 14.50 s to 3.58 s compared with basic graph-space RRT, corresponding to reductions of 68.7% and 75.3%, respectively. Ablation and statistical analyses identify goal-biased sampling as the primary contributor to improved search efficiency. Comparisons with Lifelong Planning A* across multiple start—goal configurations reveal task-dependent performance, while parameter sensitivity and simulated edge omission experiments further characterize the influence of goal bias and incomplete graph observations. These findings demonstrate the potential of RRT-based search for first-feasible-path discovery under the evaluated incremental graph-revelation conditions.

## 1. Introduction

Path planning is a fundamental problem in mobile robotics and autonomous systems, where a robot is required to find a feasible path from a start location to a target while satisfying environmental constraints. Its effectiveness depends both on the planning algorithm and how the environment is represented and made available to the planner. Conventional occupancy-based representations, such as occupancy grids [1] and OctoMap [2], primarily model the spatial occupancy of the environment. In contrast, 3D scene graphs organize geometric, topological, and semantic information within a structured representation [3]. Spatial-perception systems such as Kimera [4] and Hydra [5] have demonstrated the construction and incremental updating of 3D scene graphs from robotic sensor observations. These developments motivate path-search methods that can operate on spatial-topological information as it becomes progressively available.

The spatial-topological structure of a 3D scene graph supports graph-based path search. Classical algorithms such as Dijkstra’s algorithm [6] and A* [7] provide established solutions for known graphs, while incremental search algorithms such as Lifelong Planning A* (LPA*) [8] can reuse previous search information when the graph changes. Scene-graph-based methods have also been investigated. For example, HCOA* [9] exploits the hierarchical graph structure for path search. However, in unknown or partially observed environments, the topology available to a planner may remain incomplete as additional regions are explored. This motivates the investigation of alternative search mechanisms for path discovery over partially revealed graphs.

In this work, we consider a fixed underlying environment graph $G_{\mathrm{full}}$ and a partially revealed graph $G_{t}\subseteq G_{\mathrm{full}}$ available to the planner at iteration *t*. Starting from an initial partial graph $G_{0}$, additional nodes and edges become available through simulated local graph revelation triggered by successful search-tree expansion. The planner therefore searches for a feasible path while its available topology evolves. This formulation isolates incremental path search from online scene graph construction and physical robot execution, focusing on first-feasible-path discovery rather than shortest-path optimization over a fully known graph.

Rapidly-exploring Random Trees (RRT) offer a stochastic mechanism for incremental tree growth through sampling, nearest-node selection, and local expansion [10]. Conventional RRT operates in continuous configuration spaces, where arbitrary states may be sampled and steering generates new states. In contrast, the Places layer of a 3D scene graph [5] defines a discrete search space whose valid expansions are restricted by topological connectivity. Applying RRT to this representation therefore requires replacing continuous-state sampling with graph-node selection and reformulating steering to choose among reachable topological neighbors.

This paper proposes MSG-RRT (Multi-Strategy Scene-Graph RRT), an RRT-based method for first-feasible-path discovery over incrementally revealed 3D scene graph topology. The method operates on the Places layer using node positions and topological connectivity. Goal-biased graph-node sampling introduces target guidance while retaining stochastic exploration, whereas topology-constrained, goal-aware steering combines sample-directed and goal-directed geometric information to select the next reachable node. Newly revealed graph elements become available to subsequent sampling and tree expansion, coupling randomized search with incremental topology revelation.

MSG-RRT is evaluated on a scene graph generated from the uHumans2 [11] environment. Ablation experiments and statistical analyses examine the contributions of goal-biased sampling and goal-guided steering, while sampling-specific expansion success rates and observation gains characterize search behavior. Comparisons with LPA* across different start—goal configurations investigate task-dependent performance. Parameter sensitivity and simulated edge omission experiments further examine the effects of goal bias and incomplete observations.

The main contributions of this work are summarized as follows:

1. We formulate first-feasible-path search over incrementally revealed scene graph topology as an RRT-style tree-growth problem, coupling search-tree expansion with progressive access to place nodes and topological connections.
2. We adapt RRT-style sampling and steering to the discrete Places topology through goal-biased graph-node sampling and topology-constrained, goal-aware steering.
3. We provide a systematic experimental evaluation, including controlled ablations, statistical comparisons, an independent incremental graph-search baseline, and analyses of parameter sensitivity and incomplete observations.

## 2. Related Works

### 2.1. Sampling-Based Path Planning

Rapidly-exploring Random Trees (RRT) were introduced by LaValle [10] for sampling-based exploration of continuous state spaces. RRT repeatedly samples a state, selects the nearest node in the search tree, and extends the tree toward the sampled state. This incremental process enables exploration of large and high-dimensional configuration spaces without explicitly discretizing the entire planning domain.

Subsequent research has investigated improvements in RRT-based planning. RRT-Connect [12] grows two trees from the start and goal configurations and attempts to connect them through bidirectional expansion. RRT* [13] introduces neighborhood-based parent selection and rewiring, providing asymptotic optimality under appropriate theoretical assumptions. Further extensions have addressed planning efficiency and applicability in complex environments [14,15].

Another direction focuses on sampling and expansion strategies. Goal-biased and adaptive sampling introduce directional guidance instead of relying exclusively on uniform sampling. Informed RRT* [16] restricts sampling after an initial solution is obtained to a subset of the state space that may contain an improved solution. BIT* [17] combines sampling-based planning with heuristic graph search over increasingly dense implicit random geometric graphs. Other variants combine multiple strategies. For example, MSF-RRT [18] combines goal-biased sampling with adaptive step-size expansion. These methods illustrate how sampling and expansion strategies can influence planning efficiency in continuous spaces.

Sampling-based planning has also been combined with graph and topological representations. Rapidly-exploring Random Graphs (RRGs) construct random geometric graphs incrementally from sampled states [13]. For subterranean exploration, Dang et al. [19] combined a local rapidly-exploring random graph planner with an incrementally constructed sparse global graph. Topological maps have also been incorporated into RRT-based exploration to retain useful exploration locations and reduce repeated traversal [20].

These studies illustrate different ways of combining sampling-based planning with graph and topological representations. In RRG, the graph is constructed from sampled states in a continuous space, whereas graph-assisted exploration methods use topological structures to organize exploration and guide motion planning. These studies show how randomized planning can use topological information.

### 2.2. 3D Scene Graph Representation and Construction

3D scene graphs organize geometric, topological, and semantic information within structured environment representations. Armeni et al. [21] introduced a hierarchical 3D scene graph representation that organizes objects, rooms, and camera poses together with their spatial and semantic relationships. Such representations provide multiple levels of abstraction for robotic perception, reasoning, and planning.

Research has subsequently investigated the construction of scene graphs from robotic sensor data. Kimera [4] integrates visual-inertial estimation and metric-semantic reconstruction to support robotic spatial perception. Dynamic scene graph representations further organize places and higher-level spatial regions into hierarchical structures that can support navigation and semantic reasoning.

Hydra [5] extends this direction toward real-time, incremental scene graph construction and optimization. As a robot explores its environment, the system derives local metric information, place topology, and higher-level spatial regions from incoming observations. This demonstrates how spatial and topological information can become progressively available during robotic operation.

Recent studies have also investigated richer semantic scene representations. Strader et al. [22] investigated 3D scene graph representations for indoor and outdoor environments, while open-vocabulary approaches such as ConceptGraphs [23] incorporate language-aligned features to support flexible semantic queries. These developments expand the representational capabilities of scene graphs beyond geometric and topological information.

### 2.3. Graph and Scene-Graph-Based Planning

Graph-search algorithms provide established approaches to path planning over spatial and topological representations. Dijkstra’s algorithm [6] and A* [7] use graph connectivity and path-cost information to search for paths. Incremental heuristic search methods, such as Lifelong Planning A* (LPA*) [8], reuse previous search information when the planning graph changes. These approaches provide a basis for comparing randomized tree growth with incremental graph search.

The hierarchical structure of scene graphs has also been used for robotic planning. HCOA* [9] exploits scene graph hierarchy for path search. For long-horizon manipulation, Zhu et al. [24] introduced geometric and symbolic scene graphs as a two-level representation for visually grounded hierarchical planning, combining high-level task prediction with low-level motion generation.

Ray et al. [25] investigated task and motion planning over hierarchical 3D scene graphs. Their approach constructs sparse planning problems from relevant graph entities and progressively introduces additional objects as needed, reducing computation associated with irrelevant portions of the scene representation.

Scene graphs have also been applied to navigation in partially observed environments. Luo et al. [26] proposed an object-goal navigation framework that incrementally constructs a hierarchical scene graph from RGB-D observations and uses semantic information with learned graph-based action control. Kwon et al. [27] proposed HSG-ON, which dynamically constructs a room-workspace-object hierarchy and uses workspace-level semantic reasoning to guide exploration toward likely target locations. These studies demonstrate how incremental scene representations can support navigation and target-oriented exploration.

Existing research has established complementary approaches to planning with structured environment representations. Incremental graph-search algorithms maintain and update path-cost information as graph conditions change, while hierarchical and semantic scene graph methods exploit structured representations for planning, navigation, and task reasoning. Sampling-based methods provide randomized exploration and tree-expansion mechanisms, sometimes supported by topological maps. These developments motivate the investigation of RRT-style search directly over the discrete Places topology of a 3D scene graph. In this setting, graph nodes and connections constitute the search space itself, and their progressive revelation is coupled with randomized tree growth. The present work addresses this setting by adapting sampling and topology-constrained steering for first-feasible-path discovery.

## 3. Preliminaries

### 3.1. RRT Tree-Growth Mechanism

**Figure 1:**
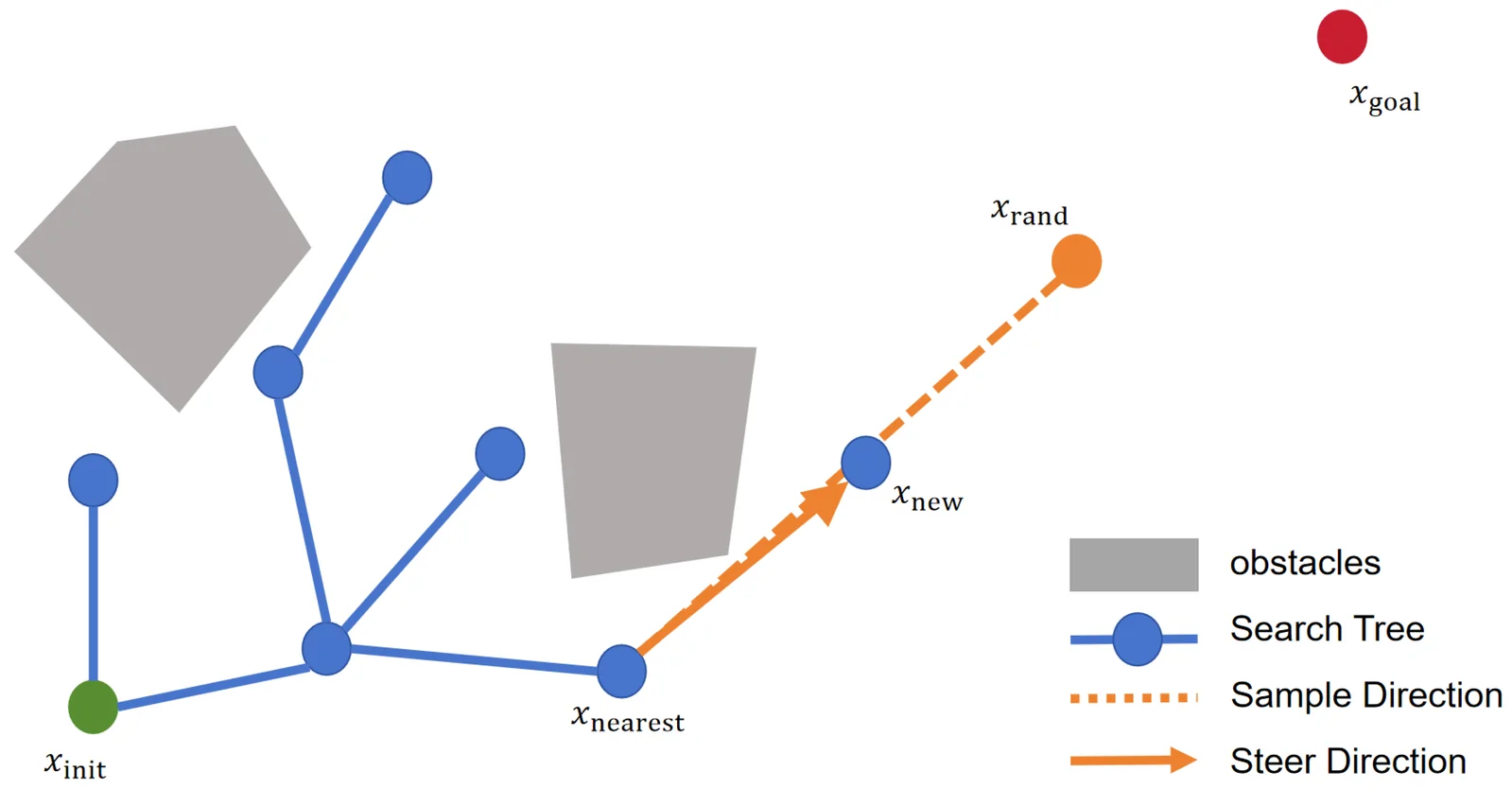
Illustration of the basic RRT tree-growth mechanism. A sampled state $x_{\mathrm{rand}}$ determines the sampling direction from the nearest tree node $x_{\mathrm{nearest}}$, while local steering generates a new state $x_{\mathrm{new}}$ toward the sample. The green and red circles represent the initial state $x_{\mathrm{init}}$ and the goal state $x_{\mathrm{goal}}$, respectively.

The Rapidly-exploring Random Tree (RRT) [10] is an incremental sampling-based planning algorithm that constructs a search tree rooted at an initial state. Let X denote the continuous configuration space and $T=(V_{T},E_{T})$ the current search tree. As illustrated in Figure 1, RRT samples a random state $x_{\mathrm{rand}}$ from the configuration space.

(1) $$x_{\mathrm{rand}}=\mathrm{Sample}\left(X\right),$$

and identifies the tree node closest to the sampled state according to a predefined distance metric d(·,·):

(2) $$x_{\mathrm{nearest}}=\arg\min_{x\in V_{T}}d\left(x,x_{\mathrm{rand}}\right).$$

A local steering operation then generates a new state from $x_{\mathrm{nearest}}$ toward $x_{\mathrm{rand}}$:

(3) $$x_{\mathrm{new}}=\mathrm{Steer}\left(x_{\mathrm{nearest}},x_{\mathrm{rand}}\right).$$

If the resulting state and local motion satisfy the feasibility and collision constraints, $x_{\mathrm{new}}$ and the corresponding edge $\left(x_{\mathrm{nearest}},x_{\mathrm{new}}\right)$ are added to the search tree. This process is repeated until the tree reaches the goal region or another termination condition is satisfied.

The sampled state determines the global tendency of tree growth, whereas nearest-node selection and local steering determine how the sample is converted into a feasible tree expansion. In conventional RRT, both sampling and steering are defined in a continuous configuration space, and $x_{\mathrm{new}}$ can be generated along a geometrically defined direction toward $x_{\mathrm{rand}}$. In the scene graph setting considered here, sampling and tree expansion operate on discrete place nodes and their topological connections. The corresponding adaptations are presented in Section 5.

### 3.2. Scene Graph Topological Representation

A 3D scene graph provides a hierarchical representation that jointly organizes geometric, topological, and semantic information about an environment [4,5]. We represent a 3D scene graph as

(4) G=(V,E,A),

where *V* denotes the graph nodes, *E* denotes intra- and inter-layer relationships, and *A* denotes node attributes such as spatial positions and semantic labels. Nodes are organized into hierarchical layers representing spatial entities at different levels of abstraction, while graph edges encode relationships such as spatial connectivity and hierarchical containment.

**Figure 2:**
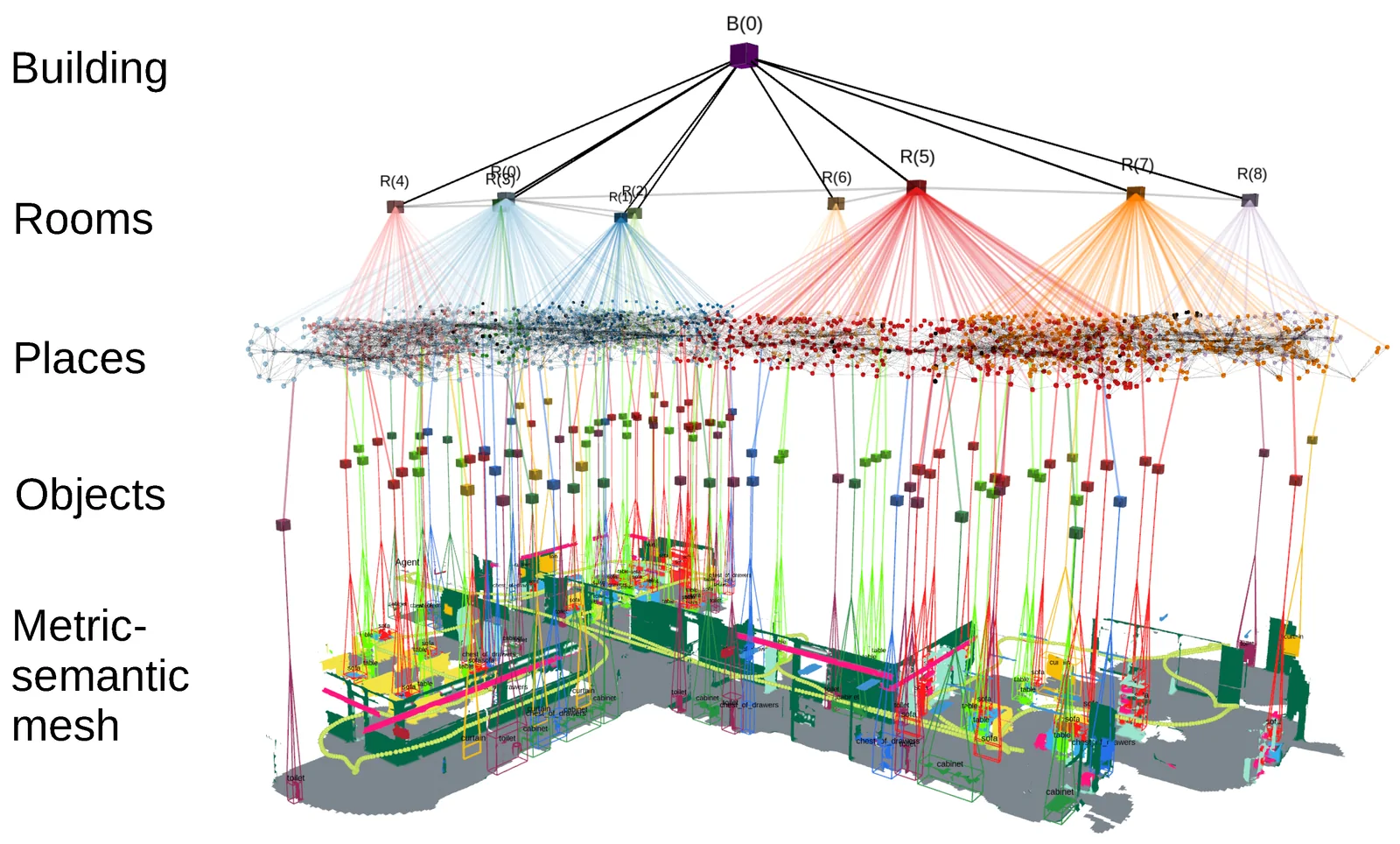
An example 3D scene graph generated by Hydra [5] using the uHumans2 office environment [11]. The place layer provides the topological search space considered in this work.

Figure 2 shows an example generated by Hydra [5] from the uHumans2 office environment [11]. Among the different layers, this work primarily uses the *Places layer* as the topological search space. Let the place-layer subgraph be denoted by

(5) $$G^{p}=\left(V^{p},E^{p},A^{p}\right),$$

where $V^{p}$ contains place nodes representing traversable spatial locations, $E^{p}$ represents local topological connectivity between these locations, and $A^{p}$ contains the associated node attributes. In particular, each place node $v\in V^{p}$ is associated with a spatial position $p\left(v\right)\in R^{3}$, which allows geometric distances and directions to be evaluated while tree expansion remains constrained by the graph topology.

This representation provides a discrete counterpart to the continuous configuration space used by conventional RRT. The Places layer thus provides the discrete search space used in this study. Section 4 introduces the distinction between the complete graph and the topology currently available to the planner.

## 4. Problem Formulation

### 4.1. Incrementally Revealed Scene Graph

Let the complete planning graph extracted from the Places layer of a 3D scene graph be denoted by  

(6) $$G_{\mathrm{full}}=\left(V_{\mathrm{full}},E_{\mathrm{full}},A_{\mathrm{full}}\right),$$

where $V_{\mathrm{full}}$ denotes the set of place nodes, $E_{\mathrm{full}}$ represents their topological connections, and $A_{\mathrm{full}}$ contains the associated node attributes, including spatial positions and available semantic information. Place nodes represent navigable spatial locations, while topological edges represent local traversability between them. The planning graph is treated as undirected, with each edge representing bidirectional connectivity between neighboring place nodes. The search process uses the spatial positions and topological connections of place nodes, whereas semantic attributes and higher-level hierarchical relations are not explicitly incorporated into the search policy.

The complete graph $G_{\mathrm{full}}$ represents the underlying environment and is not assumed to be available to the planner. Instead, the planner can access only a partially revealed graph at iteration *t*:

(7) $$G_{t}^{p}=\left(V_{t}^{p},E_{t}^{p},A_{t}^{p}\right),$$

where

(8) $$V_{t}^{p}\subseteq V_{\mathrm{full}},\;E_{t}^{p}\subseteq E_{\mathrm{full}}.$$

As new regions of the environment are observed, the available graph grows incrementally according to  

(9) $$G_{0}^{p}\subseteq G_{1}^{p}\subseteq \cdots \subseteq G_{t}^{p}\subseteq G_{\mathrm{full}}.$$

During this process, the search algorithm maintains a tree

(10) $$T_{t}=\left(V_{t}^{T},E_{t}^{T}\right),$$

where  

(11) $$V_{t}^{T}\subseteq V_{t}^{p},\;E_{t}^{T}\subseteq E_{t}^{p}.$$

Therefore, the underlying environmental graph, the currently revealed graph, and the search tree satisfy  

(12) $$T_{t}\subseteq G_{t}^{p}\subseteq G_{\mathrm{full}}.$$

This formulation differs from conventional graph-search problems in which the planning graph is assumed to be completely available before search begins. Here, the search tree is constructed while the graph representation itself is being incrementally revealed.

Given a start node $v_{s}$ and a goal node $v_{g}$, the start node belongs to the initially available graph, while the goal node belongs to the underlying complete graph:

(13) $$v_{s}\in V_{0}^{p},\;v_{g}\in V_{\mathrm{full}}.$$

The initial revealed graph $G_{0}^{p}$ is constructed from the start node $v_{s}$ and its one-hop neighborhood in $G_{\mathrm{full}}$. Thus, the initial topology available to the planner depends on the selected starting location. Subsequent graph revelation follows the *k*-hop observation rule defined below.

The goal position $p(v_{g})$ is provided as task-level prior information, but the planner does not know the goal’s topological connections in advance. The goal node may therefore be absent from the initial revealed graph, even though its position is known. A feasible connection to the goal must be discovered through incremental graph revelation. In particular, at the beginning of the search it may hold that

(14) $$v_{g}\notin V_{0}^{p}.$$

Therefore, knowing the goal location does not imply that a feasible topological path to the goal is already known. The intermediate topology required to reach the goal must be progressively revealed during search.

### 4.2. Incremental Graph Revelation

The evolution from $G_{t}^{p}$ to $G_{t+1}^{p}$ is described by a local graph revelation operator $O_{k}$, which represents the graph information made available around a selected expansion node. After a new node $v_{\mathrm{next},t}$ is added to the search tree, the newly revealed graph information is denoted by  

(15) $$\Delta G_{t}^{p}=O_{k}\left(v_{\mathrm{next},t},G_{t}^{p},G_{\mathrm{full}}\right),$$

and the currently available graph is updated according to

(16) $$G_{t+1}^{p}=G_{t}^{p}\cup \Delta G_{t}^{p}.$$

For controlled evaluation, the observation operator O is implemented using a *k*-hop graph revelation rule. Given an expansion node *v*, the locally observable node set is defined as

(17) $$V_{O}\left(v\right)=\left\{u\in V_{\mathrm{full}}|d_{G_{\mathrm{full}}}\left(u,v\right)\leq k\right\},$$

where $d_{G_{\mathrm{full}}}\left(u,v\right)$ denotes the shortest-path distance, measured in graph hops, between nodes *u* and *v* in the complete planning graph $G_{\mathrm{full}}$.

The newly revealed nodes are given by

(18) $$\Delta V_{t}^{p}=V_{O}\left(v_{\mathrm{next},t}\right)\setminus V_{t}^{p}.$$

These nodes are added to the currently available graph, together with their topological connections to other known nodes. Specifically, an edge from the complete planning graph is added when at least one of its endpoints is newly revealed and both endpoints belong to the updated node set. Previously revealed nodes and edges are retained throughout the search.

In the experiments, k=2 is used consistently for all compared methods to provide an identical local revelation condition. The complete graph is used only by the simulation environment to generate local observations; the search algorithm operates exclusively on the incrementally revealed graph and does not access unrevealed topology.

The *k*-hop revelation rule serves as an idealized graph-level abstraction of local environmental observation rather than a physical sensor model. It assumes that the nodes and topological connections within the specified neighborhood can be revealed without missed detections, omitted edges, or false connections. Sensor-dependent factors such as sensing range, field of view, occlusion, measurement noise, and localization uncertainty are not explicitly modeled. This controlled setting is adopted to evaluate the incremental search mechanism independently of a particular perception system. The effects of incomplete topology observations are additionally examined through a separate edge-omission experiment, while the default graph-revelation model remains idealized.

In the simulation, each successful tree expansion triggers local graph revelation around the newly added node. The goal condition is then checked using the updated graph $G_{t+1}^{p}$. This process does not simulate robot movement between expansion locations. In a physical system, new observations would instead depend on robot motion and sensor measurements.

### 4.3. First-Feasible-Path Search

Based on the above formulation, the considered problem is to incrementally construct a search tree from $v_{s}$ while progressively revealing the environment until a feasible connection to the goal is discovered.

To accommodate the discrete topological representation, the goal region is defined with respect to the currently revealed graph. Let $N_{t}\left(v_{g}\right)$ denote the set of place nodes directly connected to the goal through known topological edges:

(19) $$N_{t}\left(v_{g}\right)=\left\{u\in V_{t}^{p}|\left(u,v_{g}\right)\in E_{t}^{p}\right\}.$$

The currently available goal region is then defined as

(20) $$G_{t}\left(v_{g}\right)=\left\{\begin{array}{cc} \left\{v_{g}\right\}\cup N_{t}\left(v_{g}\right),\; & v_{g}\in V_{t}^{p},\\ ⌀,\; & v_{g}\notin V_{t}^{p}. \end{array}\right.$$

This definition ensures that the goal region becomes available only after the goal node and its associated topological connections have been revealed. The search terminates when a newly expanded tree node reaches the currently available goal region, indicating that the goal can be directly connected through known topology.

The first iteration satisfying this condition is defined as

(21) $$t_{\mathrm{first}}=\min\left\{t|v_{\mathrm{next},t}\in G_{t+1}\left(v_{g}\right)\right\}.$$

At each successful tree expansion, local graph revelation is performed before checking the goal condition. If the newly expanded node is the goal itself, the search terminates directly. Otherwise, if the goal has been revealed and a topological edge between the newly expanded node and the goal exists in the currently available graph, this edge is added to the search tree to complete the connection. The first feasible path is then recovered by tracing the parent relations from $v_{g}$ back to $v_{s}$. The goal node and the final connecting edge, when applicable, are included in the calculation of path node count and geometric path length.

The primary performance objective is to reduce the computational effort required to discover the first feasible path. This objective can be summarized as

(22) $$\min C_{\mathrm{search}},$$

subject to

(23) $$v_{\mathrm{next},t}\in G_{t+1}\left(v_{g}\right),$$

where $C_{\mathrm{search}}$ represents the computational effort incurred before the first feasible path is obtained. In this work, search effort is primarily evaluated using the number of search iterations and computational runtime, rather than through an explicitly optimized scalar cost function. The number of nodes and geometric length of the resulting path are additionally reported as path-quality measures, while graph coverage and observation gain are used to characterize the exploration behavior during incremental search.

The objective is to discover the first feasible path while the available graph is progressively revealed. Unlike shortest-path search on a fully known graph, the planner discovers sufficient topology to establish a connection to the goal. Path length is therefore evaluated as an outcome rather than explicitly minimized by MSG-RRT.

## 5. Methodology

Based on the incrementally revealed scene graph formulated in Section 4, this section presents the proposed MSG-RRT method for first-feasible-path search. The method first transfers the sampling and incremental tree-growth mechanisms of RRT from continuous configuration space to the discrete topological space represented by the place layer of a scene graph. On this basis, goal-biased sampling and goal-guided topological steering are introduced to reduce ineffective exploration while preserving the stochastic search behavior of RRT.

### 5.1. RRT Search in Scene Graph Topological Space

As described in Section 3, conventional RRT incrementally constructs a search tree by sampling a state from the continuous configuration space, identifying the nearest node in the existing tree, and steering toward the sampled state. In the problem considered in this work, the search space is instead the discrete topological space represented by the Places layer of the currently revealed scene graph $G_{t}^{p}=\left(V_{t}^{p},E_{t}^{p},A_{t}^{p}\right)$. Accordingly, the continuous states involved in the three core RRT operations are mapped to scene graph nodes as

(24) $$x_{\mathrm{rand}}\to v_{\mathrm{rand}},\;x_{\mathrm{nearest}}\to v_{\mathrm{nearest}},\;x_{\mathrm{new}}\to v_{\mathrm{next}}.$$

Sampling and nearest-node selection retain their original roles after replacing continuous states with graph nodes, whereas steering requires a different formulation because tree expansion is constrained by the currently revealed scene graph connectivity.

At each iteration, the basic graph-space RRT first samples a node uniformly from the currently revealed Places layer,

(25) $$v_{\mathrm{rand}}∼\mathrm{Uniform}\left(V_{t}^{p}\right).$$

Unlike continuous-space sampling, this operation selects an existing place node rather than generating an arbitrary spatial state. Nodes that have not yet been revealed are excluded from the sampling set.

Given $v_{\mathrm{rand}}$, the nearest node in the current search tree $T_{t}=\left(V_{t}^{T},E_{t}^{T}\right)$ is selected according to the Euclidean distance between place-node positions:

(26) $$v_{\mathrm{nearest}}=\arg\min_{v\in V_{t}^{T}}d\left(v,v_{\mathrm{rand}}\right),$$

where

(27) $$d\left(u,v\right)={∥p\left(u\right)-p\left(v\right)∥}_{2}.$$

Here, p(v) denotes the spatial position associated with node *v*. Euclidean distance is retained for nearest-node selection to preserve the spatial sampling behavior of RRT, rather than replacing this operation with a shortest-path search over the graph topology.

Unlike continuous-space RRT, a new state cannot be generated at an arbitrary position along the direction from $v_{\mathrm{nearest}}$ to $v_{\mathrm{rand}}$. Tree expansion must instead follow an existing edge of the currently revealed scene graph. The expandable candidate set is therefore defined as

(28) $$N_{t}\left(v_{\mathrm{nearest}}\right)=\left\{u\in V_{t}^{p}\;|\;\left(v_{\mathrm{nearest}},u\right)\in E_{t}^{p},\;u\notin V_{t}^{T}\right\}.$$

The exclusion of existing tree nodes prevents duplicate insertions, while the topological constraint ensures that each newly added tree edge belongs to the currently revealed planning graph.

To retain the directional behavior of RRT in this discrete space, the candidate neighbors are evaluated according to their consistency with the sampled node. For each candidate $u\in N_{t}\left(v_{\mathrm{nearest}}\right)$, the local expansion vector and the sampling-direction vector are defined as

(29) $$e_{u}=p\left(u\right)-p\left(v_{\mathrm{nearest}}\right),$$

and

(30) $$s=p\left(v_{\mathrm{rand}}\right)-p\left(v_{\mathrm{nearest}}\right),$$

respectively. Their directional inconsistency is measured by

(31) $$c_{\theta }^{s}\left(u\right)=\frac{1-\cos\theta_{s}\left(u\right)}{2},$$

where

(32) $$\cos\theta_{s}\left(u\right)=\frac{e_{u}^{⊤}s}{{∥e_{u}∥}_{2}{∥s∥}_{2}}.$$

Thus, $c_{\theta }^{s}\left(u\right)\in \left[0,1\right]$, with a smaller value indicating greater consistency between the candidate expansion and the sampling direction.

To handle degenerate direction vectors, a numerical tolerance $\varepsilon =10^{-8}$ is introduced. If the expansion vector has a magnitude not exceeding ε, the angular cost is set to 1. If the expansion vector is nondegenerate but the sampling-direction vector has a magnitude not exceeding ε, the angular cost is set to 0, indicating that no directional preference is imposed. Otherwise, the cosine-based cost is used, with the cosine value clipped to [−1,1] for numerical stability. The same convention is applied to goal-directed steering in Section 5.3.

The candidate-to-sample distance is normalized within the current candidate set,

(33) $${\hat{d}}_{s}\left(u\right)=\mathrm{Norm}\left(d(u,v_{\mathrm{rand}})\right),$$

where Norm(·) denotes min-max normalization over $N_{t}\left(v_{\mathrm{nearest}}\right)$. Let $d_{\min}$ and $d_{\max}$ denote the minimum and maximum distances among the candidate nodes. The normalization is defined as

(34) $$\mathrm{Norm}\left(d\right)=\left\{\begin{array}{cc} \frac{d-d_{\min}}{d_{\max}-d_{\min}},\; & d_{\max}-d_{\min}>\varepsilon ,\\ 0, & \mathrm{otherwise}. \end{array}\right.$$

When the candidate distances are approximately equal, the normalized distance term is set to zero for all candidates, since it cannot distinguish among them.

The sample-directed steering cost is then defined as

(35) $$C_{s}\left(u\right)=\alpha_{s}{\hat{d}}_{s}\left(u\right)+\left(1-\alpha_{s}\right)c_{\theta }^{s}\left(u\right),$$

where $\alpha_{s}\in \left[0,1\right]$ controls the relative contributions of distance and directional consistency. The basic graph-space RRT selects

(36) $$v_{\mathrm{next}}=\arg\min_{u\in N_{t}\left(v_{\mathrm{nearest}}\right)}C_{s}\left(u\right).$$

If the candidate set is empty, no tree expansion is performed in the current iteration, and the algorithm proceeds to the next sampling attempt.

This formulation provides a discrete counterpart to conventional RRT tree growth. Random sampling guides where the tree attempts to expand, while nearest-node selection determines the starting point of each expansion. The scene graph topology then restricts steering to existing neighboring nodes, replacing continuous state generation with discrete topological selection. This basic graph-space RRT serves as the baseline formulation upon which the goal-biased sampling and goal-guided steering mechanisms of MSG-RRT are introduced in the following subsections.

### 5.2. Goal-Biased Sampling for Incremental Search

Uniform random sampling preserves broad exploration of the available graph but does not explicitly exploit the known goal location. In an incrementally revealed environment, this may cause the search tree to spend a large number of iterations exploring regions that contribute little to reaching the goal.

As formulated in Section 4, the goal node $v_{g}$ belongs to the underlying environment graph, and its spatial position $p(v_{g})$ is provided as task-level prior information. Therefore, although the complete topological connection from the current search tree to the goal may not yet be available, the goal position can still provide directional guidance for tree growth. The goal-biased sampling strategy is defined as

(37) $$v_{\mathrm{rand}}=\left\{\begin{array}{cc} v_{g}, & q<p_{g},\\ \mathrm{Random}(V_{t}^{p}), & q\geq p_{g}, \end{array}\right.$$

where q∼U(0,1), $p_{g}\in \left[0,1\right]$ denotes the goal-sampling probability, and $\mathrm{Random}(V_{t}^{p})$ denotes uniform random sampling from the currently revealed place nodes.

When $v_{g}$ is selected, the nearest-node operation in Equation (26) identifies the search-tree node with the smallest Euclidean distance to the goal. The sampled direction used in the subsequent steering operation also points toward the goal position, thereby encouraging expansion in the target direction while respecting the revealed graph connectivity. In the remaining iterations, uniform random sampling continues to distribute expansion attempts over the currently revealed graph.

The parameter $p_{g}$ controls the trade-off between goal-directed search and stochastic exploration. A larger value increases the frequency of target-directed samples but reduces the proportion of random exploration, whereas a smaller value retains behavior closer to the basic graph-space RRT. Unless otherwise specified, $p_{g}$ is set to 0.05 in the comparative experiments. Its influence on search behavior is further examined through the sensitivity analysis in Section 6.6.

Goal-biased sampling changes the distribution of sampling targets without modifying the topological constraints on tree expansion. In contrast, goal-guided steering, introduced next, incorporates the goal position directly into the local expansion cost.

### 5.3. Goal-Guided Topological Steering

Goal-biased sampling introduces global directional preference at the sampling stage. However, even when a randomly selected node is used as $v_{\mathrm{rand}}$, the known goal location can still provide useful information for choosing among the local topological neighbors of $v_{\mathrm{nearest}}$. MSG-RRT therefore extends the sample-directed steering cost $C_{s}$ in Equation (35) by introducing an additional goal-directed term.

For each candidate node $u\in N_{t}\left(v_{\mathrm{nearest}}\right)$, the candidate-to-goal distance is normalized over the current candidate set,

(38) $${\hat{d}}_{g}\left(u\right)=\mathrm{Norm}\left(d(u,v_{g})\right),$$

where the same min-max normalization as in Equation (34) is used.

Let

(39) $$g=p\left(v_{g}\right)-p\left(v_{\mathrm{nearest}}\right)$$

denote the direction from the current nearest tree node to the goal. The directional inconsistency between the candidate expansion and the goal direction is defined as

(40) $$c_{\theta }^{g}\left(u\right)=\frac{1-\cos\theta_{g}\left(u\right)}{2},$$

where

(41) $$\cos\theta_{g}\left(u\right)=\frac{e_{u}^{⊤}g}{{∥e_{u}∥}_{2}{∥g∥}_{2}}.$$

The same numerical conventions introduced for sample-directed steering in Section 5.1 are applied when the direction vectors are degenerate.

The resulting goal-directed steering cost is

(42) $$C_{g}\left(u\right)=\alpha_{g}{\hat{d}}_{g}\left(u\right)+\left(1-\alpha_{g}\right)c_{\theta }^{g}\left(u\right),$$

where $\alpha_{g}\in \left[0,1\right]$ controls the relative contributions of goal distance and directional consistency.

The complete steering cost used by MSG-RRT is then defined as

(43) $$J\left(u\right)=w_{s}C_{s}\left(u\right)+w_{g}C_{g}\left(u\right),$$

subject to

(44) $$w_{s}\geq 0,\;w_{g}\geq 0,\;w_{s}+w_{g}=1.$$

The weights determine the relative influence of sample-directed and goal-directed guidance. In the implementation, the supplied nonnegative weights are normalized to satisfy the above constraint.

The next tree node is selected according to

(45) $$v_{\mathrm{next}}=\arg\min_{u\in N_{t}\left(v_{\mathrm{nearest}}\right)}J\left(u\right).$$

When multiple candidates attain approximately the same minimum cost, one is selected uniformly at random. An absolute tolerance of $10^{-8}$ is used to identify such candidates.

The two terms in Equation (43) play complementary roles. The sample-directed cost $C_{s}$ preserves the sample-dependent expansion behavior inherited from RRT by encouraging local expansion to follow the direction of the current sample. In contrast, the goal-directed cost $C_{g}$ introduces task-level guidance by favoring topological steps that are spatially more consistent with the known goal. The sampled node therefore determines the exploratory tendency of the current iteration, while the goal term provides additional guidance during local topological expansion. When the goal itself is sampled, the two reference directions coincide; under the default setting $\alpha_{s}=\alpha_{g}$, they yield the same local steering cost.

Both $C_{s}$ and $C_{g}$ jointly consider distance and direction. The distance terms evaluate the spatial proximity of a candidate node to the corresponding reference point, whereas the angular terms evaluate whether the local topological edge is aligned with the corresponding desired direction. This distinction is particularly important in a discrete scene graph, where the node with the smallest Euclidean distance to a reference point does not necessarily lie along the topological edge that is best aligned with that reference direction.

The steering modification does not remove the stochastic component of RRT. Goal guidance is applied only when selecting among the local topological neighbors of $v_{\mathrm{nearest}}$, after the sampled node has determined the tree node considered for expansion. Consequently, the algorithm retains sample-dependent tree growth while introducing additional goal awareness at the local decision level. Unless otherwise specified, the experiments use $\alpha_{s}=\alpha_{g}=0.5$, $w_{s}=2/3$, and $w_{g}=1/3$.

### 5.4. Incremental MSG-RRT Framework

The complete MSG-RRT framework integrates the graph-space RRT mechanism, goal-biased sampling, goal-guided topological steering, and incremental graph revelation described above.

Starting from the initial revealed graph $G_{0}^{p}$ and the start node $v_{s}\in V_{0}^{p}$, the search tree is initialized as

(46) $$T_{0}=\left(\left\{v_{s}\right\},\emptyset \right).$$

At iteration *t*, a sampled node $v_{\mathrm{rand}}$ is first generated according to Equation (37). The nearest tree node $v_{\mathrm{nearest}}$ is then determined using Equation (26). Based on the currently revealed topology, the expandable candidate set $N_{t}\left(v_{\mathrm{nearest}}\right)$ is constructed according to Equation (28).

If the candidate set is nonempty, each candidate is evaluated using the combined steering cost in Equation (43), and a minimum-cost candidate is selected as $v_{\mathrm{next},t}$. An unsuccessful expansion attempt, such as one with no valid candidate or a degenerate spatial edge, is discarded without modifying the search tree or revealing additional graph information.

For a successful expansion, the search tree is updated as

(47) $$V_{t+1}^{T}=V_{t}^{T}\cup \left\{v_{\mathrm{next},t}\right\},$$

(48) $$E_{t+1}^{T}=E_{t}^{T}\cup \left\{\left(v_{\mathrm{nearest}},v_{\mathrm{next},t}\right)\right\}.$$

Following a successful tree expansion, local graph revelation is triggered around $v_{\mathrm{next},t}$ using the observation operator defined in Section 4. The newly revealed graph information is obtained as

(49) $$\Delta G_{t}^{p}=O_{k}\left(v_{\mathrm{next},t},G_{t}^{p},G_{\mathrm{full}}\right),$$

and the currently available graph is updated according to

(50) $$G_{t+1}^{p}=G_{t}^{p}\cup \Delta G_{t}^{p}.$$

The updated graph may introduce previously unavailable place nodes and topological connections, which become available to subsequent sampling and expansion attempts. Thus, successful tree expansion and local graph revelation are coupled, allowing the search to proceed as the available topology grows incrementally.

After graph revelation, the goal condition is evaluated using the updated graph $G_{t+1}^{p}$. The search succeeds when the newly expanded node belongs to the goal region $G_{t+1}\left(v_{g}\right)$ defined in Section 4.3. If $v_{\mathrm{next},t}=v_{g}$, the goal is already included in the search tree. Otherwise, if the goal node is revealed and $\left(v_{\mathrm{next},t},v_{g}\right)\in E_{t+1}^{p}$, the goal node and the corresponding topological edge are appended to the tree.

Once the goal is reached, a feasible path π is recovered by tracing the parent relations from $v_{g}$ to $v_{s}$. The algorithm returns the first feasible path discovered, without further optimization of its length or cost. If no feasible path is found within the prescribed maximum number of search attempts, the algorithm terminates without a solution.

The complete procedure is summarized in Algorithm 1.

The resulting method preserves the fundamental RRT structure of sampling, nearest-node selection, and incremental tree growth, while adapting these operations to the discrete topology of an incrementally revealed scene graph. Goal-biased sampling introduces global task guidance by occasionally selecting the known goal as the sampling target, whereas goal-guided steering incorporates additional goal awareness into local topological expansion. Together, these mechanisms are designed to improve the efficiency of first-feasible-path discovery without requiring the complete environmental topology to be available in advance.
**Algorithm 1** MSG-RRT on an Incrementally Revealed Scene Graph**Require:** 
Planner inputs: initial graph $G_{0}^{p}$, start node $v_{s}$, goal prior $v_{g}$, goal-bias probability $p_{g}$, maximum attempts $N_{\max}$; simulation environment: underlying graph $G_{\mathrm{full}}$ for local revelation**Ensure:** 
First feasible path π, or failure
 1: $T_{0}\leftarrow \left(\left\{v_{s}\right\},\emptyset \right)$

 2: $G^{p}\leftarrow G_{0}^{p}$

 3: $T\leftarrow T_{0}$

 4: **for** 
$t=0,1,\ldots ,N_{\max}-1$ 
**do**

 5:   Sample $v_{\mathrm{rand}}$
▹ see Equation (37)

 6:   $v_{\mathrm{nearest}}\leftarrow \arg\min_{v\in V_{T}}d\left(v,v_{\mathrm{rand}}\right)$

▹ see Equation (26)

 7:   Construct $N_{t}\left(v_{\mathrm{nearest}}\right)$ using the currently revealed graph $G^{p}$

▹ see Equation (28)

 8:   **if** $N_{t}\left(v_{\mathrm{nearest}}\right)=\emptyset$ **then**

 9:     **continue**

10:   **for** $u\in N_{t}\left(v_{\mathrm{nearest}}\right)$ **do**

11:     Compute sample-directed cost $C_{s}\left(u\right)$;

▹ see Equation (35)

12:     Compute goal-directed cost $C_{g}\left(u\right)$;

▹ see Equation (42)

13:     $J\left(u\right)\leftarrow w_{s}C_{s}\left(u\right)+w_{g}C_{g}\left(u\right)$

▹ see Equation (43)

14:   $v_{\mathrm{next},t}\leftarrow \arg\min_{u\in N_{t}\left(v_{\mathrm{nearest}}\right)}J\left(u\right)$

▹ see Equation (45)

15:   **if** $p\left(v_{\mathrm{next},t}\right)=p\left(v_{\mathrm{nearest}}\right)$ **then**

16:     **continue**

17:   Add $v_{\mathrm{next},t}$ and $\left(v_{\mathrm{nearest}},v_{\mathrm{next},t}\right)$ to T

18:   $\Delta G_{t}^{p}\leftarrow O_{k}\left(v_{\mathrm{next},t},G^{p},G_{\mathrm{full}}\right)$

19:   $G^{p}\leftarrow G^{p}\cup \Delta G_{t}^{p}$

20:   **if** $v_{g}\in V\left(G^{p}\right)$ **then**

21:     **if** $v_{\mathrm{next},t}=v_{g}$ **then**

22:       $\pi \leftarrow \mathrm{RecoverPath}(T,v_{s},v_{g})$

23:       **return** π

24:     **else if** $\left(v_{\mathrm{next},t},v_{g}\right)\in E\left(G^{p}\right)$ **then**

25:       Add $v_{g}$ and $\left(v_{\mathrm{next},t},v_{g}\right)$ to T

26:       $\pi \leftarrow \mathrm{RecoverPath}(T,v_{s},v_{g})$

27:       **return** π

28: **return** failure


### 5.5. Computational Complexity Analysis

The computational cost of the core MSG-RRT search operations mainly consists of graph-node sampling, nearest-node search, and topology-constrained steering. The following analysis focuses on these operations and separates their computational cost from incremental graph revelation and other simulation-related processing. Therefore, the derived bounds characterize the core search procedure rather than the complete execution time of the simulation.

The sampling procedure selects either the goal node with probability $p_{g}$ or a randomly sampled node from the currently revealed Places-layer graph. Assuming that graph nodes can be randomly accessed, this operation requires constant time, i.e.,

(51) $$T_{\mathrm{sample}}=O\left(1\right).$$

Given a sampled node $v_{\mathrm{rand}}$, the current implementation determines $v_{\mathrm{nearest}}$ by evaluating its Euclidean distance to all nodes already contained in the search tree. For a tree with $|V_{T}|$ nodes, the corresponding complexity is

(52) $$T_{\mathrm{nearest}}=O(|V_{T}\left|\right).$$

The steering procedure subsequently constructs the expandable candidate set by examining the topological neighbors of $v_{\mathrm{nearest}}$. Let $\deg_{t}\left(v_{\mathrm{nearest}}\right)$ denote the degree of this node in the currently revealed graph $G_{t}^{p}$. Constructing the candidate set requires examining at most $\deg_{t}\left(v_{\mathrm{nearest}}\right)$ neighbors.

For each candidate $u\in N_{t}\left(v_{\mathrm{nearest}}\right)$, MSG-RRT evaluates the sample-directed cost $C_{s}$, the goal-directed cost $C_{g}$, and their weighted combination. Since these operations involve a constant number of distance and directional calculations in a fixed-dimensional space, evaluating each candidate requires constant time. The candidate evaluation cost is therefore $O(|N_{t}\left(v_{\mathrm{nearest}}\right)|)$. Since $|N_{t}\left(v_{\mathrm{nearest}}\right)|\leq \deg_{t}\left(v_{\mathrm{nearest}}\right)$, the overall steering complexity is

(53) $$T_{\mathrm{steer}}=O\left(\deg_{t}\left(v_{\mathrm{nearest}}\right)\right).$$

Consequently, the computational complexity of the core operations in one search attempt is

(54) $$T_{\mathrm{iter}}=O\left(|V_{T}|+\deg_{t}\left(v_{\mathrm{nearest}}\right)\right).$$

Since the Place layer of a 3D scene graph is typically represented as a locally connected sparse topological graph, the number of neighboring nodes is generally much smaller than the number of nodes accumulated in the search tree. Under this condition, the nearest-node search constitutes the dominant growing term in the core search procedure.

Let *K* denote the total number of search attempts before termination, including unsuccessful expansions. The cumulative complexity of the core search operations is

(55) $$T_{\mathrm{search}}=O\left(\sum_{k=1}^{K}\left(|V_{T}^{\left(k\right)}|+\deg_{k}\right)\right),$$

where $V_{T}^{\left(k\right)}$ denotes the search-tree node set at attempt *k*, and $\deg_{k}$ denotes the degree of the selected nearest node in the currently revealed graph.

Since each successful expansion adds at most one new node to the tree, while unsuccessful attempts leave the tree unchanged, $|V_{T}^{\left(k\right)}|\leq k+1$. Moreover, if the maximum degree of the revealed graph is bounded by Δ, then $\deg_{k}\leq \Delta$. An upper bound for the core search procedure is therefore

(56) $$T_{\mathrm{search}}=O\left(K^{2}+K\Delta \right).$$

For a sparse graph with bounded local degree, this reduces to $O(K^{2})$, primarily due to the linear nearest-node search performed at each attempt. This bound characterizes the current core search implementation rather than an inherent limitation of the proposed search formulation. Spatial indexing or dedicated nearest-neighbor data structures may further reduce the nearest-search overhead for larger graphs.

## 6. Evaluation

### 6.1. Experimental Setup

The proposed method is evaluated using the uHumans2 dataset [11]. The place layer of the generated 3D scene graph serves as the topological search space, consisting of 2091 nodes and 8365 edges. The complete place graph is retained by the simulation environment as $G_{\mathrm{full}}$, whereas the planner is initialized with a locally revealed subgraph $G_{0}^{p}$. During search, additional nodes and edges are incrementally incorporated into the currently known graph $G_{t}^{p}$ according to the local revelation mechanism described in Section 5. A fixed two-hop revelation range (k=2) is used throughout the experiments.

Three locations, denoted by A, B, and C, are selected from the Places layer to construct different start—goal tasks. Location A corresponds to the starting region of the original scene graph construction process. Location B is a distant target whose reference shortest path on the complete graph generally progresses toward the goal. Location C is geometrically closer to A, but the route from A to C requires a topological detour, including segments that temporarily move away from the goal. Three tasks are considered: A—B, A—C, and C—A. The reversed C—A task is included to examine how the starting region and search direction affect incremental path discovery. For each task, the initial revealed graph consists of the start node and its one-hop neighbors. All compared methods use the same initial graph, revelation rule, and termination criteria within each task. With start location A, the initial graph contains 16 nodes and 45 edges, while there are 2 nodes and 1 edge with start location C.

All experiments are conducted on a laptop equipped with an Intel Core i9-14900HX processor, an NVIDIA GeForce RTX 4090 GPU, and 64 GB RAM, using Python 3.8.10. All RRT-based configurations are subject to a maximum of 10,000 search iterations, which was not reached in any of the evaluated runs. Each stochastic configuration was evaluated over 20 independent runs. For run index i=0,…,19, the random seed was set to 42+i, and both Python’s random module and NumPy’s random-number generator were initialized with this seed before the corresponding run. Thus, the experiments used seeds from 42 to 61. All reported runs successfully discovered a feasible path within the iteration limit. Results are presented as the mean ± sample standard deviation, with 95% confidence intervals provided where appropriate.

To isolate the contributions of goal-biased sampling and goal-guided topological steering, four configurations are considered, as summarized in Table 1.

RRT denotes the basic graph-based implementation introduced in Section 5.1, employing uniform random sampling and sample-directed steering. G-RRT incorporates goal-biased sampling while retaining the baseline steering rule, whereas S-RRT introduces goal-guided steering while maintaining uniform random sampling. MSG-RRT combines both strategies. Unless otherwise specified, the goal-sampling probability is fixed at $p_{g}=0.05$.

The primary evaluation metrics are the number of search iterations and the total computational runtime required to discover the first feasible path. Search iterations count all executions of the main RRT search loop, including unsuccessful expansion attempts. Runtime measures the wall-clock execution time of the complete search procedure, including sampling, steering, tree expansion, and simulated graph revelation, but excluding post-run evaluation.

Graph-revelation calls count the local revelation operations triggered after successful tree expansions. Final graph coverage is defined as

(57) $$C_{\mathrm{final}}=\frac{|V_{t}^{p}|}{|V_{\mathrm{full}}|},$$

where $V_{t}^{p}$ and $V_{\mathrm{full}}$ denote the place-node sets of the revealed graph at termination and the complete graph, respectively. Path-node count and path length characterize the resulting feasible paths, with path length including the final connection to the goal node. Success rate is defined as the proportion of runs that successfully discover a feasible path within the iteration limit. Sampling-specific expansion success rates and observation gains are additionally examined to characterize the effects of different search strategies.

For the ablation study, two-sided paired *t*-tests are performed on runs with corresponding random seeds to assess differences between configurations. The primary comparisons consider search iterations and runtime, while path length is examined as a secondary metric. The resulting *p*-values are adjusted using the Holm procedure to account for multiple comparisons, with a significance level of 0.05.

### 6.2. Incremental Search Demonstration

**Figure 3:**
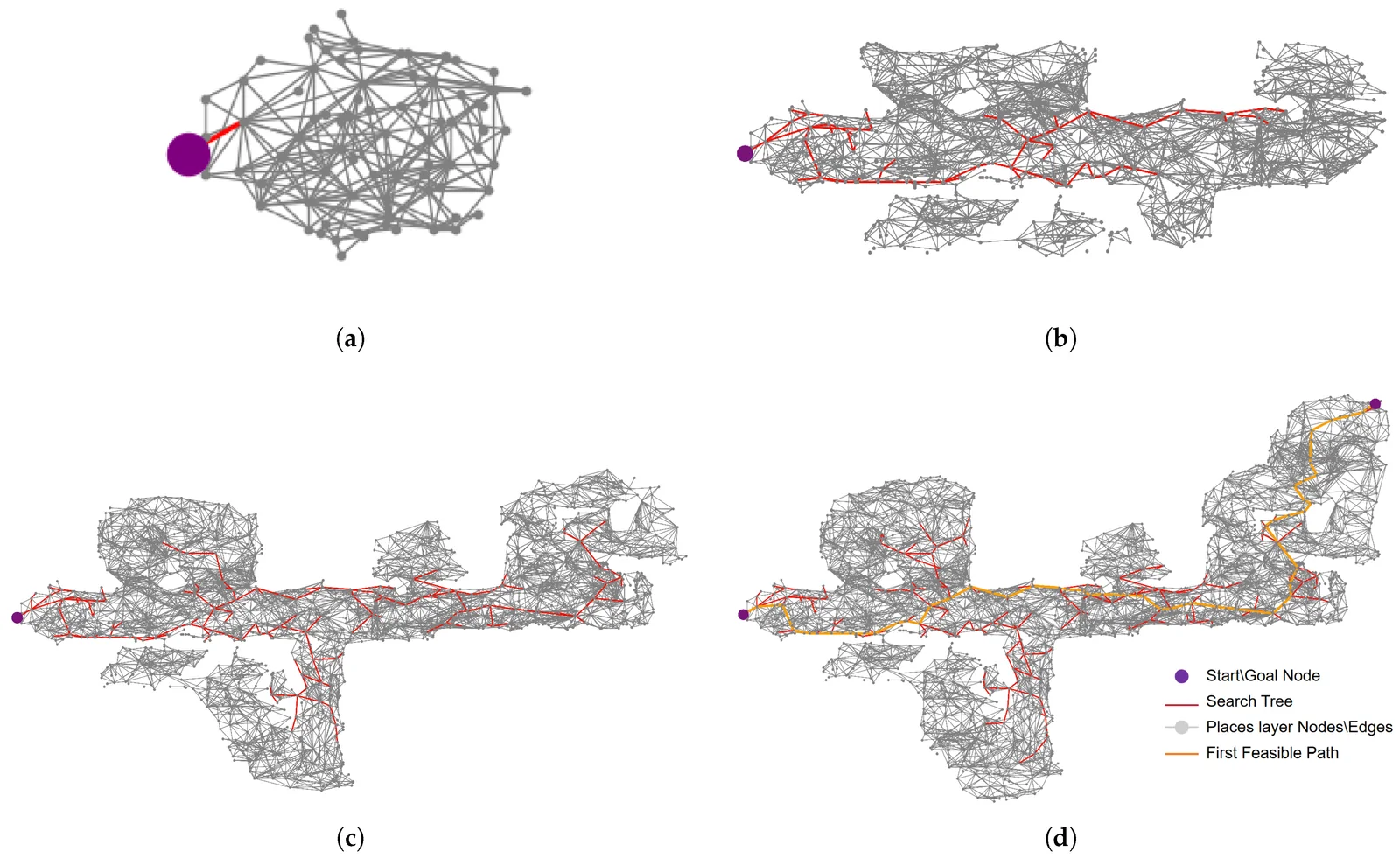
Representative incremental search process of MSG-RRT. (**a**) Initialization; (**b**,**c**) intermediate search states; (**d**) first feasible path found. Gray nodes and edges represent the revealed scene graph, red edges represent the search tree, purple markers indicate the start and goal, and the orange line highlights the recovered path.

Figure 3 illustrates a representative MSG-RRT search run under incremental graph revelation. The search starts with a local portion of the place-layer topology around the start node. As the search tree expands, the observation operator described in Section 4 reveals additional graph neighborhoods, making new nodes and edges available for subsequent search iterations. The four panels show the progression from initialization to the discovery of the first feasible path.

This example illustrates how MSG-RRT interleaves search-tree expansion with local graph revelation. Newly revealed topology becomes available for subsequent expansion decisions, while the search tree remains restricted to the currently known graph. The resulting path is therefore discovered incrementally, without requiring the complete scene-graph topology to be available at initialization.

### 6.3. Search Performance and Ablation Study

Table 2 summarizes the search performance of the four configurations introduced in Table 1. The ablation study evaluates the individual and combined effects of goal-biased sampling and goal-guided topological steering under the same incremental graph-revelation conditions. All configurations successfully discovered feasible paths in the 20 independent runs.

The baseline RRT requires an average of 2185.45 iterations and 14.50 s to discover a feasible path. Introducing goal-biased sampling alone substantially reduces the required search effort. G-RRT decreases the average number of iterations to 806.35 and runtime to 4.46 s, corresponding to reductions of 63.1% and 69.3%, respectively. Goal-guided steering also improves search efficiency when applied independently. S-RRT reduces the average iteration count to 1681.45 and runtime to 11.08 s, corresponding to reductions of 23.1% and 23.6%. These results indicate that both modifications can improve search efficiency, although goal-biased sampling produces the larger reduction under the evaluated conditions.

Combining the two strategies yields the lowest average search effort among the four configurations. MSG-RRT requires 683.90 iterations and 3.58 s on average, reducing the iteration count and runtime by 68.7% and 75.3%, respectively, relative to the baseline RRT. Compared with G-RRT, MSG-RRT further reduces the average iteration count by 15.2% and runtime by 19.7%. However, these additional reductions must be interpreted in conjunction with the statistical analysis rather than solely from their numerical differences.

To assess the statistical support for the observed improvements, two-sided paired *t*-tests are conducted using runs with corresponding random seeds. The comparisons examine search iterations, runtime, and path length, with *p*-values adjusted using the Holm procedure across the 15 tests. Table 3 summarizes the results for the two primary search-efficiency metrics.

The statistical results confirm the substantial contribution of goal-biased sampling. Both RRT-to-G-RRT and S-RRT-to-MSG-RRT comparisons show statistically significant reductions in iterations and runtime ($p_{\mathrm{adj}}<0.05$). Goal-guided steering also produces statistically significant improvements when introduced independently, as demonstrated by the RRT-to-S-RRT comparison. However, the additional reductions observed when steering is combined with goal-biased sampling (G-RRT versus MSG-RRT) do not reach statistical significance for either metric. Therefore, while the combined configuration achieves the lowest average search effort, the present results provide stronger statistical support for the contribution of goal-biased sampling than for the additional efficiency gain of steering in the combined configuration.

**Figure 4:**
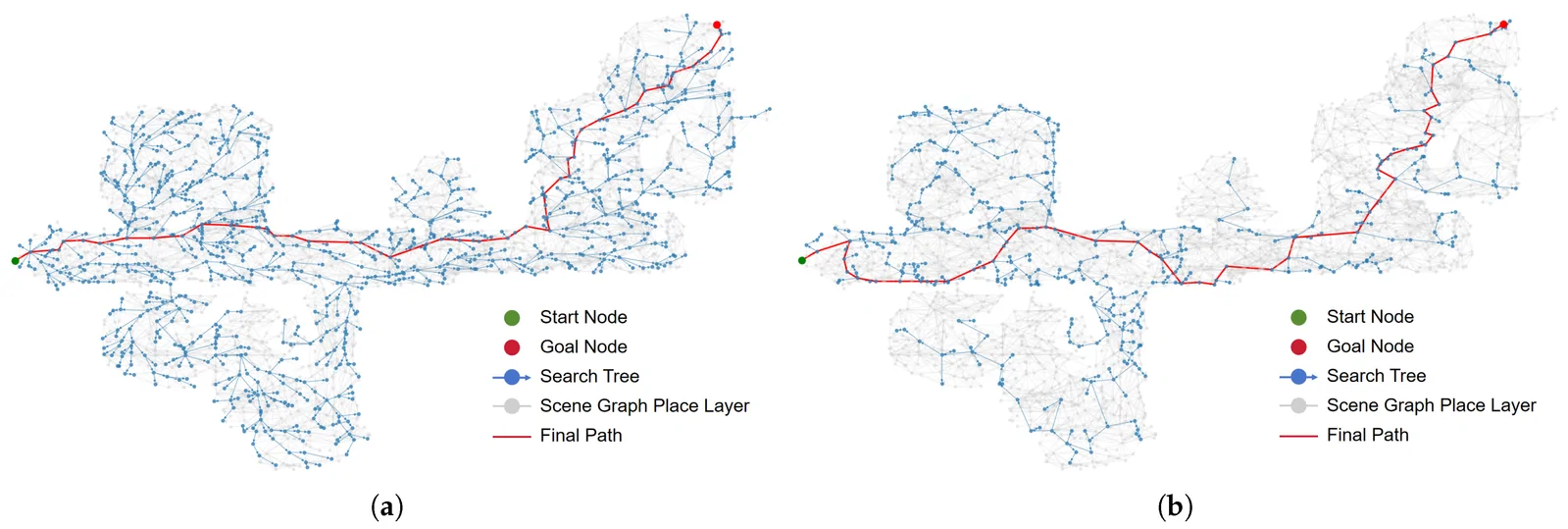
Example search-tree structures generated by (**a**) the baseline RRT and (**b**) MSG-RRT for the same start—goal configuration. Gray nodes and edges indicate the revealed scene graph topology, blue nodes and edges indicate the search tree constructed before termination, and the red line denotes the first feasible path.

Figure 4 illustrates the differences in tree-growth behavior between the baseline RRT and MSG-RRT. In the illustrated baseline run, the search tree extends over a larger portion of the revealed topology before reaching the goal, whereas MSG-RRT terminates with a more spatially concentrated tree. The figure provides a qualitative example of how sampling and steering guidance can influence tree growth. The corresponding reduction in search effort is established quantitatively by the repeated-run results in Table 2 and Table 3, rather than by the visualization alone.

The observed runtime reduction is consistent with the computational analysis in Section 5.5. Although goal-guided steering introduces additional local candidate evaluations, MSG-RRT requires fewer search iterations under the evaluated conditions, resulting in a lower overall runtime. This suggests that the reduction in search attempts can offset the additional steering cost.

The reduction in search effort is accompanied by lower graph coverage at termination. The baseline RRT reveals an average of 94.77% of the place nodes, whereas MSG-RRT terminates at an average coverage of 78.32%. Since graph revelation is triggered by successful tree expansion and the search terminates upon discovering the first feasible path, lower final coverage is consistent with the reduced amount of exploration required before termination. This result does not indicate greater environmental exploration capability; rather, it reflects the different search effort required to accomplish the same path-discovery task.

The resulting path characteristics provide additional information about the solutions obtained by each configuration. MSG-RRT returns paths with an average length of 75.36 m, compared with 82.18 m for the baseline RRT, corresponding to a reduction of 8.3%. This difference is statistically significant after Holm correction ($p_{\mathrm{adj}}=0.0101$). Goal-biased sampling alone also produces a statistically significant reduction in path length relative to RRT ($p_{\mathrm{adj}}=0.0116$). In contrast, the path-length differences associated with introducing goal-guided steering do not reach statistical significance after correction. Thus, the observed path-length reduction of the complete method should be interpreted as an empirical characteristic of the returned feasible paths, without attributing a statistically established independent path-quality improvement to the steering strategy. MSG-RRT is designed for first-feasible-path discovery rather than shortest-path optimization, and no optimality guarantee is implied by these results.

### 6.4. Comparison with Incremental Graph Search

**Figure 5:**
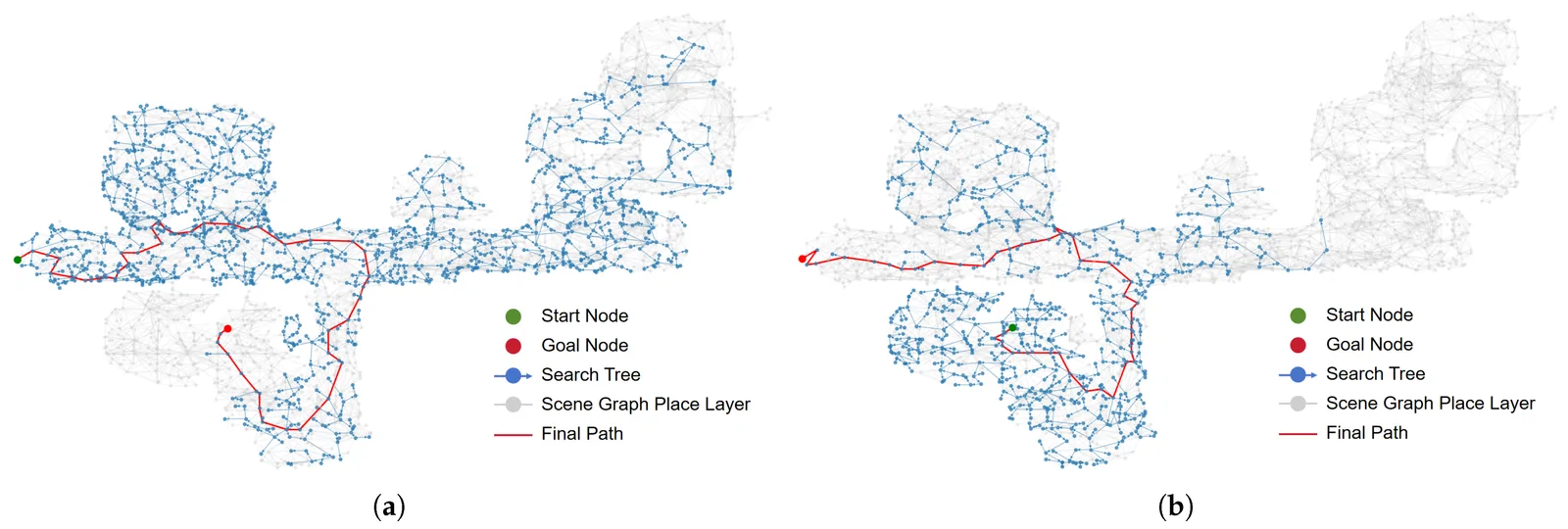
Example MSG-RRT search outcomes for the reversed start—goal configurations: (**a**) A—C and (**b**) C—A. Gray nodes and edges represent the complete place-layer graph, blue nodes and edges denote the topology revealed by termination, and red lines indicate the returned first feasible paths. The endpoint markers identify the start and goal locations. Although the two configurations involve the same pair of nodes, reversing the search direction changes the initial local topology and the subsequent graph-revelation process.

To further evaluate the proposed method against an independent incremental graph-search baseline, MSG-RRT is compared with Lifelong Planning A* (LPA*) [8]. Both methods use the same initial revealed graph and the same local graph-revelation rule for each start—goal configuration. However, their different search mechanisms lead to different revelation sequences and numbers of observation calls. Consequently, graph-revelation calls are reported to characterize search behavior rather than as directly equivalent measures of computational work. MSG-RRT employs randomized sampling and goal-guided steering to discover a first feasible path, whereas LPA* incrementally updates its path-cost estimates as the known graph changes. The comparison uses the A—B, A—C, and C—A tasks defined in Section 6.1. A—B examines long-range search with relatively consistent geometric and topological guidance, whereas A—C examines a route requiring a detour away from the goal. C—A reverses the latter task to assess the effect of search direction and the start-dependent initial graph. The corresponding search examples are shown in Figure 4b and Figure 5a,b.

As shown in Table 4, both methods successfully discover feasible paths in all evaluated runs. In the long-range A—B configuration, MSG-RRT achieves an average runtime of 3.58 s, compared with 4.44 s for LPA*. MSG-RRT also requires fewer graph-revelation calls, although its final graph coverage is higher. In contrast, LPA* returns a shorter path of 58.59 m, whereas MSG-RRT produces paths with an average length of 75.36 m. These results show that randomized first-feasible-path search can achieve competitive computational performance in this configuration, but does not provide the same path-length performance as the incremental graph-search baseline.

The relative performance changes considerably in the A—C configuration. Although the reference path is shorter than that of A—B, the feasible route includes segments that temporarily increase the geometric distance to the target. MSG-RRT requires an average of 1180.30 iterations and 6.66 s, whereas LPA* completes the search in 1.77 s. The latter also requires fewer graph-revelation calls and produces a shorter path. The goal-sampling expansion success rate of MSG-RRT decreases from 84.77% in A—B to 25.02% in A—C. This observation is consistent with the reduced effectiveness of direct goal-directed expansion when the available topological route requires a geometric detour.

Reversing the search direction from A—C to C—A also leads to a substantial change in search behavior. Although both configurations have the same reference shortest-path length of 38.53 m on the complete graph, the average MSG-RRT runtime decreases from 6.66 s to 2.88 s, while its average number of search iterations decreases from 1180.30 to 826.30. LPA* exhibits a similar directional difference in computational effort, with average runtimes of 1.77 s and 0.54 s for A—C and C—A, respectively. These observations suggest that search difficulty under incremental graph revelation is not determined by the final path length alone.

Figure 5 illustrates the different search outcomes of the A—C and C—A configurations. The initial known topology is determined by the neighborhood of the start node. In this environment, node C has a smaller local neighborhood with fewer connections than node A. Consequently, reversing the start and goal changes the initially available expansion choices and the order in which additional topology becomes accessible. The two examples exhibit different spatial patterns of graph revelation and path discovery, consistent with the differences in runtime and revelation calls reported in Table 4. The visualization provides a qualitative illustration of this direction-dependent behavior rather than isolating a single causal factor.

Overall, goal-biased sampling produces higher observation gains per goal-directed attempt, while goal-guided steering influences the success and outcome of local expansions. These results help explain the differences in search effort observed in the ablation study.

### 6.5. Search Behavior Analysis

To further investigate the mechanisms underlying the performance differences observed in Section 6.3, the search behavior of the four configurations is analyzed using the same experimental runs as the ablation study. The analysis focuses on expansion success rates and the number of newly revealed place nodes associated with different sampling strategies.

An expansion is considered successful when a sampling attempt results in a successful tree expansion and triggers a local graph-revelation operation. The expansion success rate is defined as the ratio of successful expansions to the number of sampling attempts of the corresponding type. Observation gain measures the number of newly revealed place nodes following an expansion. To account for both successful and unsuccessful sampling attempts, observation gain per sample is calculated by dividing the total number of newly revealed nodes associated with a sampling type by its total number of attempts.

As shown in Table 5, the baseline RRT achieves a random-sampling expansion success rate of 67.51%, with an average observation gain of 0.94 newly revealed nodes per sampling attempt. Introducing goal-guided steering alone increases these values to 74.02% and 1.19 for S-RRT, respectively. In comparison, G-RRT achieves a random-sampling expansion success rate of 86.12% and an observation gain of 2.01 nodes per random sample. MSG-RRT further increases these values to 88.26% and 2.12, respectively. These differences indicate that the sampling and steering configurations affect not only the number of iterations required for path discovery but also the behavior of individual expansion attempts.

For the configurations employing goal-biased sampling, goal-directed samples account for only a small proportion of the total search attempts. Nevertheless, they produce substantially larger observation gains per sample than random samples. In MSG-RRT, goal-directed sampling yields an average of 9.78 newly revealed nodes per attempt, compared with 2.12 for random sampling. A similar difference is observed in G-RRT, with respective gains of 9.58 and 2.01. These results show that goal-directed sampling reveals more previously unknown place nodes per attempt in this configuration.

Importantly, the larger observation gain associated with goal-directed sampling does not necessarily correspond to a higher expansion success rate. In MSG-RRT, the goal-sampling expansion success rate is 84.77%, slightly lower than the random-sampling success rate of 88.26%. Thus, the contribution of goal-biased sampling cannot be explained solely by increasing the probability of successful expansion. Instead, the recorded observation gains indicate that successful goal-directed expansions tend to reveal more previously unknown place nodes, thereby influencing the growth of the available topology during search.

The influence of goal-guided steering is more modest. Compared with the baseline RRT, S-RRT achieves higher random-sampling expansion success and observation gain per sample. Similarly, MSG-RRT exhibits slightly higher values than G-RRT. These observations are consistent with the steering criterion influencing local neighbor selection, although the behavioral differences alone do not establish an additional statistically significant reduction in total search effort when goal-biased sampling is already enabled, as discussed in Section 6.3.

Overall, the search-behavior analysis complements the performance ablation by showing how goal-directed sampling and local steering affect successful tree expansion and incremental graph revelation. In the evaluated configuration, goal-biased sampling is associated with substantially greater topology revelation per goal-directed attempt, while steering modifies the behavior of local expansions. These findings provide a behavioral interpretation of the observed search-efficiency differences without implying that greater graph revelation necessarily leads to shorter paths or universally better performance.

### 6.6. Sensitivity to Goal-Bias Probability

**Figure 6:**
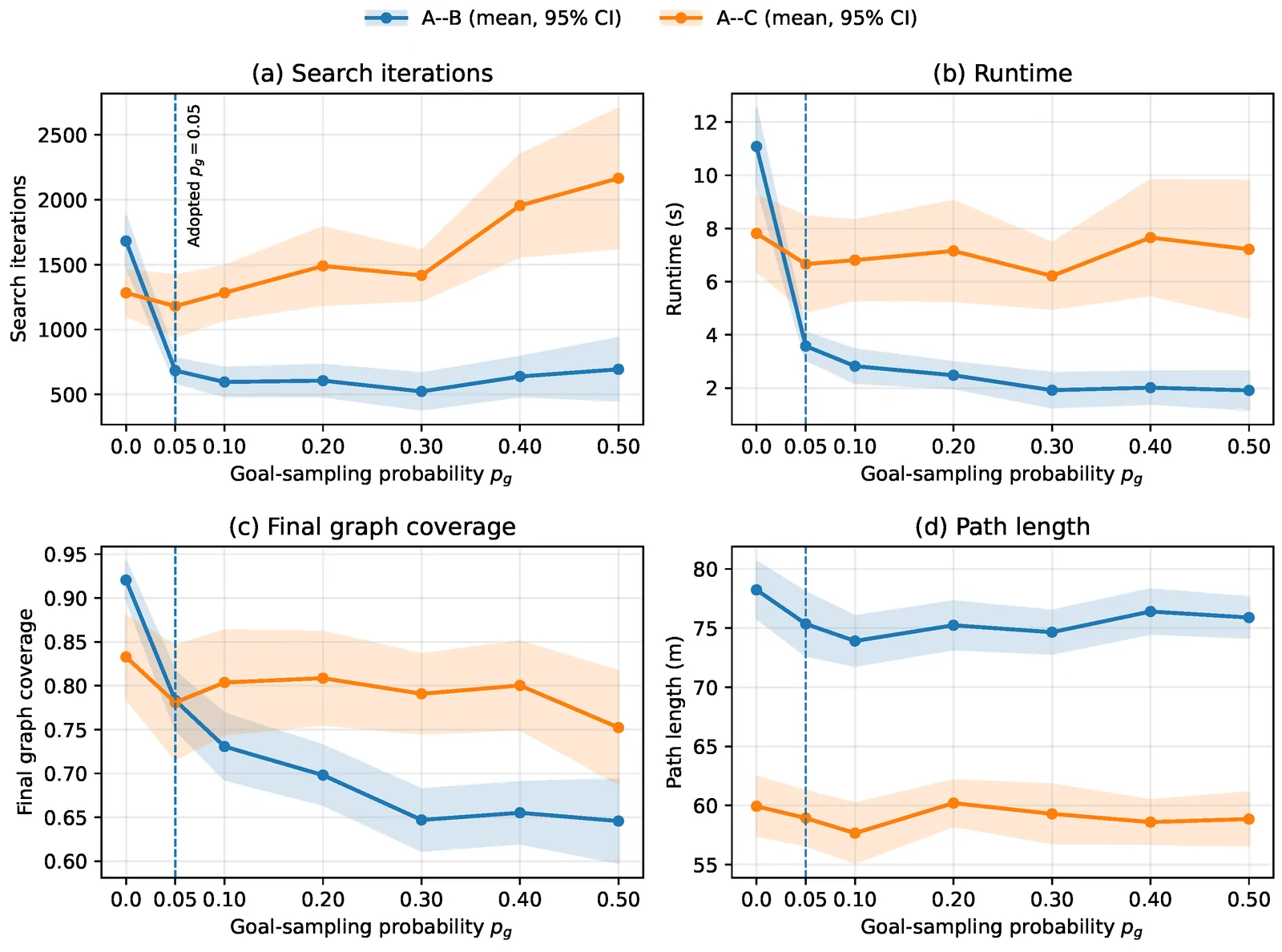
Sensitivity of MSG-RRT to the goal-sampling probability $p_{g}$ under the A—B and A—C configurations: (**a**) search iterations, (**b**) runtime, (**c**) final graph coverage, and (**d**) path length. Each curve represents the mean over 20 runs, and the shaded band indicates the 95% confidence interval of the mean. The dashed vertical line marks the adopted default setting $p_{g}=0.05$.

To evaluate the influence of goal-biased sampling, the goal-sampling probability $p_{g}$ is varied over {0,0.05,0.1,0.2,0.3,0.4,0.5}, while the goal-guided steering strategy and other experimental settings remain unchanged. Two start—goal configurations are considered: A—B, representing a long-range search task, and A—C, in which reaching the goal requires a topological detour. Each setting is evaluated over 20 independent runs. Table 6 reports the search and path statistics, and Figure 6 illustrates the corresponding trends.

For the A—B configuration, introducing a small goal-sampling probability substantially reduces search effort. Increasing $p_{g}$ from 0 to 0.05 decreases the average number of iterations from 1681.45 to 683.90 and runtime from 11.08 s to 3.58 s, corresponding to reductions of approximately 59.3% and 67.7%, respectively. Further increases in $p_{g}$ maintain relatively low search effort, although the changes are not monotonic. The lowest mean iteration count is obtained at $p_{g}=0.3$, while the lowest mean runtime is observed at $p_{g}=0.5$. Thus, the results do not indicate that $p_{g}=0.05$ is the optimal setting for this configuration.

In contrast, the effect of goal-biased sampling is less pronounced for the A—C configuration. Increasing $p_{g}$ from 0 to 0.05 reduces the average iteration count from 1282.50 to 1180.30 and runtime from 7.81 s to 6.66 s. However, the iteration count generally increases at larger probabilities, reaching 2165.70 at $p_{g}=0.5$. The runtime exhibits substantial variation and does not follow a strictly monotonic trend. Since the A—C task requires traversing topological connections that temporarily move away from the target, frequent goal-directed sampling may be less effective in supporting the exploration needed to discover a feasible route.

The sampling-specific statistics in Table 7 provide further insight into these differences. For A—B, the goal-sample expansion success rate decreases from 84.77% at $p_{g}=0.05$ to 20.98% at $p_{g}=0.5$, while the corresponding observation gain decreases from 9.78 to 2.61 newly revealed nodes per sample. In comparison, the random-sample expansion success rate remains approximately 88–90% over this range. Goal-directed sampling therefore provides a greater observation gain at low probabilities, but its effectiveness per attempt decreases as goal sampling becomes more frequent.

For A—C, goal-directed sampling is less effective even at low probabilities. At $p_{g}=0.05$, the goal-sample expansion success rate is 25.02%, compared with 79.00% for random sampling. The corresponding observation gains are 0.95 and 1.57 nodes per sample, respectively. As $p_{g}$ increases to 0.5, the goal-sample success rate decreases to 2.17%, and its observation gain falls to 0.14 nodes per sample. These results indicate that frequent goal-directed sampling may contribute relatively little to tree expansion and graph revelation when the feasible route requires a topological detour.

The effects on the returned paths are less systematic. For A—B, the average path-node count ranges from 47.80 to 51.00, while the average path length ranges from 73.91 m to 78.24 m. For A—C, these ranges are 39.35–41.05 nodes and 57.66–60.20 m, respectively. As shown in Table 6, $p_{g}=0.05$ does not consistently yield the fewest path nodes or the shortest path. Moreover, the variations in path characteristics are relatively small compared with their standard deviations. The preferred goal-sampling probability therefore depends on the task and evaluation metric.

The final graph coverage also varies with $p_{g}$, as illustrated in Figure 6c. For A—B, coverage generally decreases as goal-biased sampling becomes more frequent, consistent with earlier termination after fewer expansions. In A—C, the coverage trend is less regular, reflecting the more variable search process. Since MSG-RRT terminates upon discovering the first feasible path, lower final coverage does not necessarily indicate poorer search performance. Overall, the results show that the effectiveness of goal-biased sampling depends on the relationship between geometric target guidance and the available graph topology. A small nonzero probability retains random exploration while introducing occasional goal-directed expansion attempts. Accordingly, $p_{g}=0.05$ is used as a conservative default, while the sensitivity results indicate that the preferred setting depends on the task.

### 6.7. Robustness to Incomplete Graph Observations

To investigate the influence of incomplete topological observations, an edge-omission mechanism is introduced into the incremental graph-revelation process. During each local revelation operation, newly observable edges are independently omitted with probability $p_{\mathrm{missing}}$. Previously revealed edges are retained, while omitted edges remain absent from the revealed graph because the observation procedure only introduces connections associated with newly revealed nodes. The underlying complete graph remains unchanged. Four omission probabilities, $p_{\mathrm{missing}}\in \left\{0,0.1,0.2,0.3\right\}$, are evaluated using the A—B configuration. The sampling and steering parameters remain unchanged, with $p_{g}=0.05$. Each setting is evaluated over 20 independent runs. This experiment considers missing topological edges rather than node omissions or false-positive connections.

The edge-omission experiments use an additional graph-revelation procedure that copies and merges graph updates to simulate missing connections. When $p_{\mathrm{missing}}=0$, this procedure reveals the same nodes and edges as the standard implementation, but may produce different neighbor traversal orders. Since steering randomly selects among near-optimal candidates, these ordering differences can lead to different search trajectories and numerical results. As shown in Table 8, MSG-RRT successfully discovers feasible paths in all evaluated runs, including those with edge-omission probabilities up to 0.3. Nevertheless, incomplete graph observations generally increase the search effort. Compared with $p_{\mathrm{missing}}=0$, the average number of search iterations increases from 662.80 to 958.70 at $p_{\mathrm{missing}}=0.2$, corresponding to an increase of approximately 44.6%. The average number of graph-revelation calls also increases from 576.10 to 724.60, while runtime rises from 6.72 s to 8.06 s.

The observed changes are not strictly monotonic. At $p_{\mathrm{missing}}=0.3$, the average iteration count decreases slightly to 915.95, compared with 958.70 at $p_{\mathrm{missing}}=0.2$. However, both configurations exhibit substantial variability, with standard deviations exceeding 400 iterations. Their confidence intervals also overlap considerably. Therefore, this small difference should not be interpreted as an improvement in search efficiency at a higher omission probability. Rather, the results indicate that the search effort varies with the particular topology revealed during randomized exploration.

The returned path characteristics also show moderate changes under incomplete observations. The average path length increases from 75.62 m without edge omissions to 79.93 m at $p_{\mathrm{missing}}=0.2$, before decreasing slightly to 78.85 m at $p_{\mathrm{missing}}=0.3$. Final graph coverage remains within approximately 77.7–80.1% across the tested settings. Since graph revelation and path discovery are coupled, changes in final coverage reflect both the missing connections and the amount of search performed before termination. Higher coverage therefore does not necessarily indicate more accurate observations or better search performance.

Overall, the results suggest that MSG-RRT can continue to discover feasible paths under the evaluated levels of random edge omission, although the required search effort and its variability generally increase. This experiment provides a limited assessment of robustness to incomplete topological connectivity. It does not address missing place nodes, false-positive edges, localization uncertainty, or errors in the geometric positions of graph nodes. The observed success rates characterize performance under the tested edge-omission model.

## 7. Discussion

The ablation results show that goal-directed guidance can reduce the search effort required for first-feasible-path discovery over incrementally revealed scene-graph topology. Goal-biased sampling accounts for most of the improvement, while goal-guided steering also provides benefits when used independently. However, the additional improvement obtained by combining the two strategies is not statistically significant compared with goal-biased sampling alone. This suggests that sampling guidance plays a more important role in the evaluated setting. By increasing the frequency of goal-directed expansion attempts, goal bias can help the search tree reach useful regions without exploring as much of the available graph. Goal-guided steering, in contrast, influences the selection of neighboring nodes during individual expansions.

The sampling-behavior and sensitivity results provide further insight into the role of goal bias. In the A—B task, goal-directed samples produce relatively high observation gains when the goal-sampling probability is small. As the probability increases, the success rate and observation gain per goal sample decline, although successful goal-directed expansions can still reveal substantial new topology. In the A—C task, goal-directed sampling is less effective even at low probabilities, while random sampling maintains a higher expansion success rate. This difference is consistent with the topological detour required in A—C, where progress along a feasible route may temporarily increase the Euclidean distance to the goal. These observations suggest that the usefulness of goal bias depends on how well geometric goal guidance agrees with the available graph connectivity. An adaptive sampling strategy could adjust this balance during search.

The comparison with LPA* further illustrates the influence of task topology and search strategy. MSG-RRT achieves a lower average runtime in the long-range A—B task, whereas LPA* returns a shorter path. In the A—C and C—A tasks, LPA* achieves both lower runtime and shorter paths. Both methods use the same initial graph and local revelation rule, but their different search procedures lead to different observation sequences. MSG-RRT grows a randomized search tree and stops when a feasible connection to the goal is found. LPA*, meanwhile, maintains path-cost information as new graph elements become available. The results suggest that randomized first-feasible-path search can be efficient when goal-directed expansion provides useful guidance, while incremental graph search can be preferable when reaching the goal requires substantial movement away from its geometric direction. The resulting path length is also an important consideration when choosing between the methods.

The edge-omission experiments examine the effect of incomplete topological observations. MSG-RRT successfully finds feasible paths in all tested runs, while the number of search attempts and its variability generally increase under edge omissions. In the adopted model, each newly observable edge may be omitted during graph revelation, while previously revealed edges are retained. Since the revelation procedure introduces edges only when new nodes are added, omitted connections are not automatically recovered by later observations. Consequently, the experiments evaluate the effect of persistent missing connections in the revealed graph rather than repeated sensor measurements that may recover missed observations. Missing nodes, false connections, and uncertain node positions remain relevant conditions for further evaluation.

An important consideration for robotic deployment is the relationship between search-tree expansion and physical observation. In the current simulation, a successful tree expansion triggers local graph revelation around the newly added node. This allows the search mechanism to be studied without simulating robot movement between successive expansion locations. In a physical system, however, new graph information would become available only after the robot moves to an observation location and acquires sensor measurements. Consequently, the planner would need to coordinate tree expansion with robot execution, maintain feasible routes to observation locations, and incorporate graph updates obtained from the perception system. Sensor visibility and localization uncertainty would also affect which nodes and edges become available. Integrating these processes with incremental scene-graph systems is an important direction for future development.

Finally, the evaluation is based on one uHumans2 scene graph with several start—goal configurations, and the observation range is fixed at two graph hops. Although each tree expansion follows a single topological edge, a larger observation radius can reveal more surrounding connections and affect subsequent search decisions. The influence of this parameter on search efficiency remains to be examined. The current search policy uses positions and topological connections from the Places layer, without explicitly exploiting semantic attributes or higher-level scene-graph hierarchy. The experimental results therefore characterize graph-space search under controlled revelation conditions. Further evaluation on different environments and graph structures would help establish how search performance changes with topology and scale. Future work will also investigate adaptive sampling, execution-aware graph updates, and tighter integration with online scene-graph construction.

## 8. Conclusions

This paper presented MSG-RRT, an RRT-based method for first-feasible-path search over incrementally revealed 3D scene graph topology. The method adapts sampling and steering to the discrete Places layer, combining goal-biased sampling with topology-constrained, goal-aware expansion.

Experiments on the evaluated uHumans2 scene graph demonstrate that MSG-RRT reduces the average search iterations from 2185.45 to 683.90 and computational runtime from 14.50 s to 3.58 s compared with the basic graph-space RRT, corresponding to reductions of 68.7% and 75.3%, respectively. The ablation and statistical analyses indicate that goal-biased sampling provides the primary efficiency improvement, while goal-guided steering also improves search performance when applied independently. However, its additional contribution in the combined configuration is not statistically significant under the evaluated conditions. Comparisons with incremental LPA* further reveal that MSG-RRT achieves a lower average runtime in the long-range task, whereas LPA* performs better in the detour-related configurations and returns shorter paths. The goal-bias sensitivity analysis shows that search efficiency depends on the relationship between geometric guidance and topological connectivity. Experiments with randomly omitted topological edges additionally show successful feasible-path discovery under the tested omission probabilities, although search effort generally increases.

The results demonstrate the potential of randomized tree search over incrementally revealed spatial-topological graphs, with performance depending on task topology and observation conditions. Future work will extend the evaluation to additional environments and investigate adaptive sampling, execution-aware graph updates, and integration with online robotic perception.

## Figures and Tables

**Table 1:** Configurations used in the ablation study. A ✓ indicates an enabled strategy, while a × indicates a disabled strategy.

Method	Goal-Biased Sampling	Goal-Guided Steering
RRT	×	×
G-RRT	✓	×
S-RRT	×	✓
MSG-RRT	✓	✓

**Table 2:** Search performance of different sampling and steering configurations (mean ± standard deviation over 20 runs).

Method	Iterations	Runtime (s)	Coverage (%)	Path Nodes	Path Length (m)
RRT	2185.45±514.30	14.50±3.45	94.77±2.73	55.40±5.14	82.18±5.23
G-RRT	806.35±331.61	4.46±2.55	81.69±8.24	48.55±3.98	76.21±4.59
S-RRT	1681.45±406.19	11.08±3.05	92.04±4.47	51.00±4.90	78.24±5.09
MSG-RRT	683.90±196.51	3.58±1.07	78.32±7.00	48.75±5.40	75.36±5.70

**Table 3:** Statistical comparison of search efficiency between ablation configurations. Relative changes are calculated with respect to the baseline of each comparison. Adjusted *p*-values are obtained using the Holm procedure.

Comparison	Iteration Change (%)	Adjusted *p*	Runtime Change (%)	Adjusted *p*
RRT → G-RRT	−63.10	$1.62\times 10^{-7}$	−69.28	$1.29\times 10^{-7}$
RRT → S-RRT	−23.06	0.0116	−23.60	0.00837
G-RRT → MSG-RRT	−15.19	0.558	−19.74	0.558
S-RRT → MSG-RRT	−59.33	$1.32\times 10^{-7}$	−67.73	$6.54\times 10^{-8}$
RRT → MSG-RRT	−68.71	$1.24\times 10^{-8}$	−75.34	$2.18\times 10^{-9}$

**Table 4:** Comparison of MSG-RRT and incremental LPA* under three start—goal configurations. Results are reported as mean ± standard deviation over 20 runs. Deterministic values are reported without standard deviations.

Task	Method	Runtime (s)	Revelation Calls	Coverage (%)	Path Length (m)
A—B	MSG-RRT	3.58±1.07	593.05±134.38	78.32±7.00	75.36±5.70
	LPA*	4.44±0.18	967	67.29	58.59
A—C	MSG-RRT	6.66±3.83	871.30±301.62	78.09±13.79	58.93±4.91
	LPA*	1.77±0.11	669	45.72	38.53
C—A	MSG-RRT	2.88±1.46	571.10±148.24	65.22±10.17	60.55±7.02
	LPA*	0.54±0.05	407	33.09	38.53

**Table 5:** Sampling-specific expansion success rates and observation gains in the ablation study (mean ± standard deviation over 20 runs). N/A indicates that the corresponding sampling strategy is not used.

Method	Random Success (%)	Goal Success (%)	Random Gain/Sample	Goal Gain/Sample
RRT	67.51±6.83	N/A	0.94±0.19	N/A
G-RRT	86.12±5.87	74.11±19.64	2.01±0.66	9.58±4.56
S-RRT	74.02±6.65	N/A	1.19±0.24	N/A
MSG-RRT	88.26±5.05	84.77±12.74	2.12±0.40	9.78±3.00

**Table 6:** Sensitivity to the goal-sampling probability under the A—B and A—C configurations. Results are reported as mean ± standard deviation over 20 runs.

Task	$p_{g}$	Iterations	Runtime (s)	Path Nodes	Path Length (m)
A—B	0.00	1681.45±406.19	11.08±3.05	51.00±4.90	78.24±5.09
	0.05	683.90±196.51	3.58±1.07	48.75±5.40	75.36±5.70
	0.10	595.75±234.28	2.82±1.34	47.80±3.97	73.91±4.45
	0.20	606.25±259.37	2.48±1.04	48.30±3.51	75.24±4.32
	0.30	522.90±296.85	1.92±1.37	48.80±2.95	74.65±3.85
	0.40	638.35±322.91	2.01±1.29	49.60±4.39	76.40±3.99
	0.50	693.50±513.73	1.91±1.53	49.75±3.67	75.89±3.64
A—C	0.00	1282.50±373.52	7.81±3.03	39.35±4.48	59.93±5.29
	0.05	1180.30±503.99	6.66±3.83	39.75±3.51	58.93±4.91
	0.10	1283.25±439.88	6.81±3.19	39.70±3.96	57.66±5.34
	0.20	1489.85±637.80	7.15±4.02	41.05±2.76	60.20±4.13
	0.30	1416.90±408.25	6.22±2.64	39.90±3.73	59.29±5.30
	0.40	1955.40±836.73	7.66±4.62	40.15±3.66	58.59±3.95
	0.50	2165.70±1148.98	7.22±5.50	40.55±4.08	58.85±4.81

**Table 7:** Sampling-specific expansion success rates and observation gains under different goal-sampling probabilities. Values are means over 20 runs. N/A indicates that the corresponding goal-directed sampling metric is not applicable when $p_{g}=0$.

Task	$p_{g}$	Expansion Success (%)		Gain per Sample	
		Goal	Random	Goal	Random
A—B	0.00	N/A	74.02	N/A	1.19
	0.05	84.77	88.26	9.78	2.12
	0.10	65.25	88.16	7.93	2.29
	0.20	39.96	88.66	4.66	2.25
	0.30	36.08	89.97	4.65	2.70
	0.40	23.19	89.35	2.68	2.37
	0.50	20.98	88.48	2.61	2.76
A—C	0.00	N/A	77.96	N/A	1.44
	0.05	25.02	79.00	0.95	1.57
	0.10	14.22	78.25	0.52	1.51
	0.20	6.15	77.52	0.32	1.49
	0.30	4.92	82.09	0.26	1.64
	0.40	2.62	78.10	0.16	1.46
	0.50	2.17	77.84	0.14	1.55

**Table 8:** Performance of MSG-RRT under different edge-omission probabilities (mean ± standard deviation over 20 runs). All configurations achieved 20/20 successful runs.

$p_{\mathrm{missing}}$	Iterations	Runtime (s)	Revelation Calls	Coverage (%)	Path Length (m)
0.0	662.80±167.77	6.72±2.21	576.10±130.18	77.68±6.14	75.62±3.78
0.1	762.70±283.10	7.08±3.62	630.25±195.45	78.03±8.40	77.60±5.83
0.2	958.70±455.18	8.06±4.43	724.60±267.36	80.08±9.67	79.93±6.06
0.3	915.95±422.57	7.10±4.31	686.90±248.71	79.40±7.73	78.85±5.52

## Data Availability

The original contributions presented in this study are included in the article. Further inquiries can be directed to the corresponding author.
